# Analgesic Effect of Moxibustion with Different Temperature on Inflammatory and Neuropathic Pain Mice: A Comparative Study

**DOI:** 10.1155/2017/4373182

**Published:** 2017-10-09

**Authors:** Wei Zhou, Ruxue Lei, Chuanyi Zuo, Yunqing Yue, Qin Luo, Chengshun Zhang, Peng Lv, Yong Tang, Haiyan Yin, Shuguang Yu

**Affiliations:** ^1^Acupuncture and Tuina School, Chengdu University of Traditional Chinese Medicine, Chengdu, Sichuan 610075, China; ^2^Department of Rehabilitation Medicine, People's Hospital of Deyang City, Deyang, Sichuan 618000, China

## Abstract

The aim of this study was to determine whether variation of temperature during moxibustion would generate division of analgesic effect. The moxibustion with different temperatures (37°C, 42°C, 47°C, and 52°C) was applied to ST36 acupoint for 30 minutes in chronic inflammatory or neuropathic pain mice. The analgesic effect was evaluated by thermal hyperalgesia test in chronic inflammatory pain and by mechanical allodynia in neuropathic pain, respectively. The results indicated that interventions of moxibustion with different temperature caused different analgesic effect on either chronic inflammatory induced by injection of complete Freund's adjuvant (CFA) or neuropathic pain induced by spared nerve injury (SNI). In chronic inflammatory pain, different moxibustion temperature generated different intensity of analgesic effect: the higher the better. In chronic neuropathic pain, stronger analgesic effect was found in moxibustion with temperature 47°C or 52°C other than 37°C and 42°C. However, there is no significant difference displayed between moxibustion temperatures 47°C and 52°C or 37°C and 42°C. It implies that the temperature should be taken into account for moxibustion treatment to chronic inflammatory or neuropathic pain.

## 1. Introduction

Chronic pain, including chronic inflammatory and neuropathic pain, has becoming a crucial health threat all over the world [1]. Seeking out an effective approach to relieving pain has been a focus in pain management research. Moxibustion, as an important member in acupuncture family procedures [2], has demonstrated that it could be employed to reduce the pain intensity in different types of pain caused by a variety of diseases [3–7]. Moxibustion therapy is manipulated with ignited mugwort (*Artemisia vulgaris* from traditional Chinese medicine) directly or indirectly at acupoints or some specific parts of body [8]. Generally speaking, one session of moxibustion stimulation lasts about 30 minutes. In the course of moxibustion treatment, the temperature has been widely recognized as a key factor to affect moxibustion curative effect [9–13]. Clinical and experimental studies have also indicated that moxibustion needs proper moxibustion temperature to achieve better therapeutic effect [14–16]. However, which temperature would be better to produce analgesic effect remains unclear. Therefore, we proposed a hypothesis that different temperature moxibustion would give rise to different analgesic effect in different pattern of pain and designed the experiment to address this issue.

## 2. Materials and Methods

### 2.1. Animals

Male C57BL/6J mice weighing 20 ± 2 g were purchased from the Beijing HuaFuKang Bioscience Co., Ltd. All mice were acclimatized to standard laboratory conditions (24 ± 2°C room temperature, 65 ± 5% humidity, on 12/12h light-dark cycles) with drinking water and food available ad libitum. The experimental procedures were conducted in accordance with the National Institutes of Health (NIH) Guide for the Care and Use of Laboratory Animals [17] and approved by the Animal Ethics Committee of Chengdu University of Traditional Chinese Medicine.

After adaptive domestication for one week, mice were firstly randomly divided into two groups: chronic inflammatory pain group and neuropathic pain group, based on random numbers generated by SPSS software. Then, the mice in chronic inflammatory pain group were randomly divided into the following six subgroups: control group, CFA group, CFA-moxi-37°C group, CFA-moxi-42°C group, CFA-moxi-47°C group, and CFA-moxi-52°C group (*n* = 18 each group). The mice in chronic neuropathic pain group were randomly divided into control (sham surgery) group, SNI group, SNI-moxi-37°C group, SNI-moxi-42°C group, SNI-moxi-47°C group, and SNI-moxi-52°C group (*n* = 14 each group).

### 2.2. Chronic Inflammatory and Neuropathic Pain Model

Chronic inflammatory pain model was induced by subcutaneously injecting 20 *μ*L complete Freund's adjuvant (CFA, Sigma, USA) into the right hind paw [18]. In control group, mice were injected 20 *μ*L normal saline (KeLun Industry Group, Sichuan, China) instead of CFA. In this study, thermal hyperalgesia was detected at the fourth day (pain peak time) after CFA injection [19].

Neuropathic pain model was established by spared nerve injury (SNI) [20]. All mice were deeply anaesthetized with isoflurane and were shaved on the right side below the pelvis. The thigh was incised through the skin and then muscle to expose the sciatic nerve and its three terminals branches: the sural, peroneal, and tibial nerves. The peroneal and tibial nerves were isolated from surrounding fascia and were tightly ligated with silk and transected distal to the ligation. Sham operated mice served as controls and underwent the same procedure but did not receive any nerve manipulation. In all surgery groups, the muscle and skin layers were closed separately using absorbable suture, and mice were allowed to recover and got intraperitoneal injection of penicillin (80000 U/kg, North China Pharmaceutical Factory, China) for 3 days to prevent infection. Mechanical hyperalgesia was detected at the fourth day after SNI surgery to assess neuropathic pain model.

### 2.3. Intervention

**Figure 1:**
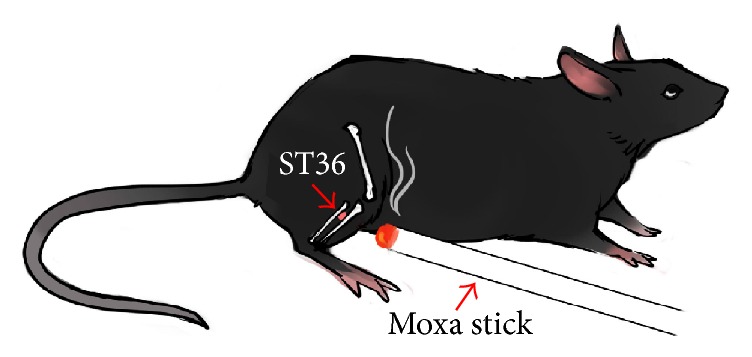
Schematic diagram of ST36 location and moxibustion intervention.

**Figure 2:**
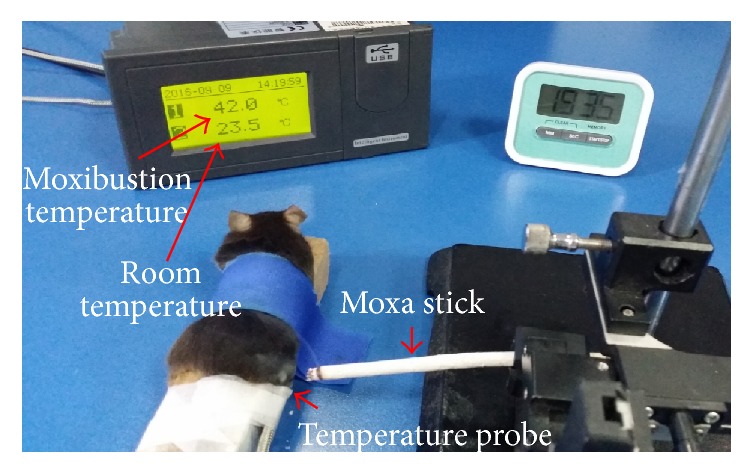
Moxibustion intervention.

**Figure 3:**
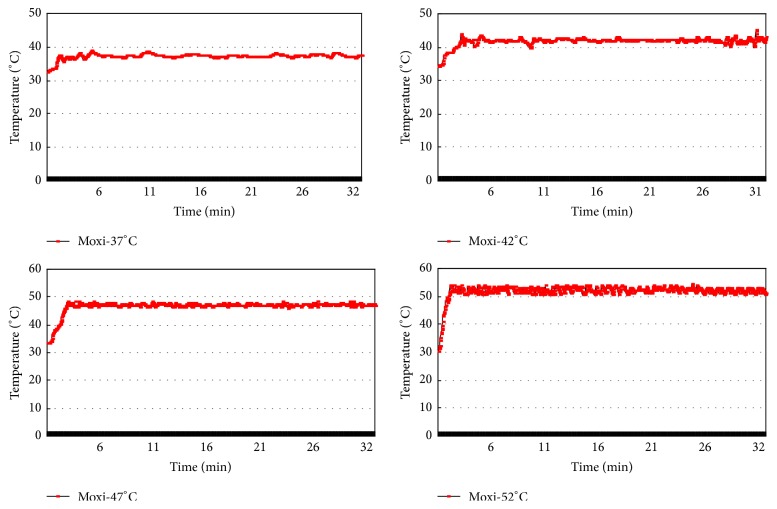
Temperature curves on ST36 during moxibustion.

Moxibustion was performed at the fourth day after CFA injection and SNI surgery. Moxibustion temperature was set at 37°C, 42°C, 47°C, and 52°C with an interval of 5°C. Before moxibustion, fur on the outer lateral surface of right hind limb around ST36 (Zusanli acupoint, located at the posterolateral knee of hind limbs, about 2 mm below the fibular head [21], Figure 1) was shaved to expose ST36. Animal-used moxa sticks (diameter × length: 8 mm × 20 cm, Nanyang Hanyi Moxibustion Technology Development Co., Ltd., China) were burned to carry out moxibustion over the right ST36 for 30 min (Figure 1). Mice in control and model group without moxibustion intervention were restricted for 30 min. Moxibustion temperature of ST36 was monitored by a digital thermodetector (WZ-2300R, Xingyi Electronics Company, Hangzhou, China) and stabilized at 37 ± 1°C, 42 ± 1°C, 47 ± 1°C, and 52 ± 1°C (Figures 2 and 3).

### 2.4. Measurement of Pain Threshold

#### 2.4.1. Thermal Withdrawal Latency

**Figure 4:**
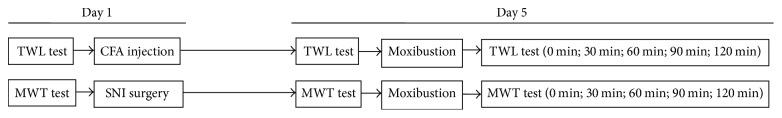
Flow chart of the experiment.* Notes*. TWL and MWT were tested on day 1 before modeling, on day 5 before moxibustion intervention, and at the 0 min, 30 min, 60 min, 90 min, and 120 min after moxibustion intervention.

Thermal hyperalgesia was assessed by measuring the thermal withdrawal latency (TWL) with a Plantar Test Apparatus (Hargreaves method, PL-200, Tai Meng, China). Mice were placed in behavioral boxes on a glass platform [22]. After 30 min of acclimatization, a mobile radiant heat source (a high-intensity light beam of radiant heat dolorimeter) was focused on the plantar surface of right hind paw. Light intensity was preset at 10%, in order to obtain a baseline latency of ~10 sec and the cutoff time was set at 20 sec to avoid tissue damage. Any of following three responses to thermal stimuli was taken as threshold of pain [23]: (1) the velocity of the withdrawal reflex; (2) the presence or absence of licking; and (3) the duration of the hind paw withdrawal from the floor. The paw TWL was obtained three times per animal with intervals of 5 min. The average from three measurements was calculated as the final result of pain threshold. Paw TWL was tested three times per animal with intervals of 5 min on day 1 before CFA injection and day 5 before moxibustion, while it was tested only once at the 0 min, 30 min, 60 min, 90 min, and 120 min after moxibustion intervention separately (Figure 4).

#### 2.4.2. Mechanical Withdrawal Threshold

Mechanical hyperalgesia was assessed by detecting mechanical withdrawal threshold (MWT) with a dynamic plantar aesthesiometer (37450, UGO Basile, Germany) [24–27] and expressed in grams. Briefly, the mice were individually placed in the transparent acrylic box under the wire mesh bench, allowing access to the plantar surface of the hind paw. After 20 min of acclimation, gentle incremental pressure (from 0 to 25 g over a 10 s period) was applied using a rigid von Frey hair (0.5 mm diameter) to the plantar surface of the ipsilateral hind paw, until the paw was withdrawn. Three tests were conducted at intervals of 5 min and the force (*g*) applied was recorded. MWT was averaged from three measurements (before SNI surgery and after SNI surgery). Each mouse was, respectively, trained for this test on day 1 before SNI surgery, on day 5 before the moxibustion intervention, and at the 0 min, 30 min, 60 min, 90 min, and 120 min after moxibustion. MWT was measured only once each mouse at five time points after moxibustion intervention. The paw MWL was tested three times per animal with intervals of 5 min on day 1 before CFA injection and day 5 before moxibustion, while it was tested only once at the 0 min, 30 min, 60 min, 90 min, and 120 min after moxibustion intervention separately (Figure 4).

### 2.5. Statistical Analysis

All data were analyzed and graphed using Graphpad Prism6 (GraphPad Software, Inc., La Jolla, CA, USA). Data were presented as means ± SEM (standard error of means). Comparisons among groups were performed by two-way analysis of variance (ANOVA), followed by Tukey's post hoc testing for comparisons between control and experimental groups. A value of *P* < 0.05 was considered statistically significant.

## 3. Results

### 3.1. Thermal Withdrawal Latency

**Figure 5:**
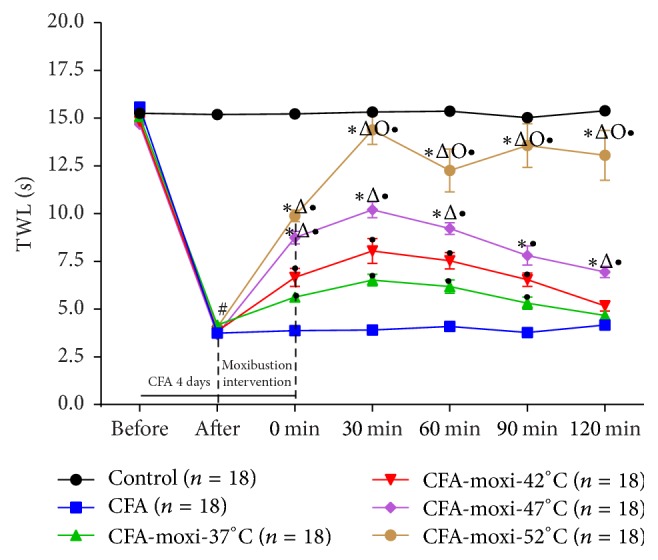
Effect of moxibustion on pain threshold of CFA mice. ^#^*P* < 0.05 versus control group; ^•^*P* < 0.05 versus CFA group; ^*∗*^*P* < 0.05 versus CFA-moxi-37°C group; ^Δ^*P* < 0.05 versus CFA-moxi-42°C group; ^Ο^*P* < 0.05 versus CFA-moxi-47°C group.

As shown in Figure 5 and Table 1, the TWL-pain threshold in control group nearly did not display any change (*P* > 0.05) at any test point, while after CFA injection, the TWL of CFA group and moxibustion groups was decreased remarkably (*P* < 0.05). Compared with model group, all the four moxibustion groups demonstrated a clear evidence of TWL increasing (*P* < 0.05) at 0 min, 30 min, 60 min, and 90 min after moxibustion, and all TWL arrived their peaks at 30 min after moxibustion. Regarding the difference among four different temperatures, TWL of CFA-moxi-52°C group was notably higher (*P* < 0.05) than any other moxibustion group, even at 120 min after moxibustion. And it demonstrated that the higher the moxibustion temperature, the better the analgesic effect (CFA-moxi-37°C < CFA-moxi-42°C < CFA-moxi-47°C < CFA-moxi-52°C).

### 3.2. Mechanical Withdrawal Threshold

**Figure 6:**
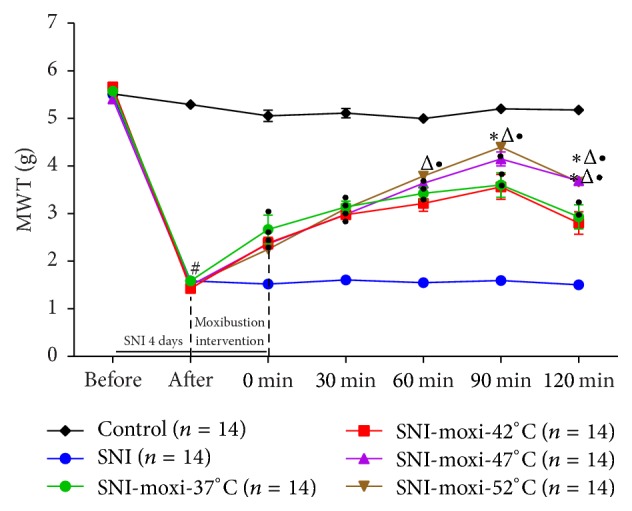
Effect of moxibustion on pain threshold of SNI mice. ^#^*P* < 0.05 versus control group; ^•^*P* < 0.05 versus SNI group; ^*∗*^*P* < 0.05 versus SNI-moxi-37°C group; ^Δ^*P* < 0.05 versus SNI-moxi-42°C group.

As shown in Figure 6 and Table 2, the MWT of control group nearly did not display any change as time went on (*P* > 0.05), while it was decreased distinctly (*P* < 0.05) in SNI model group. Compared with model group, the MWT of each moxibustion intervention groups was significantly increased (*P* < 0.05) and peaked at 90 min after moxibustion. Comparing the four different moxibustion groups, a trend at 60 min after moxibustion that the higher moxibustion temperature, the higher MWT could be seen. At 90 min and 120 min after moxibustion, the MWT of 47°C and 52°C moxibustion groups were obviously higher than 37°C and 42°C moxibustion groups (*P* < 0.05), but there was no statistically significant difference between 52°C and 47°C group (*P* > 0.05).

## 4. Discussion

Moxibustion temperature was well recognized as a key factor to produce curative effect in moxibustion therapy [6–10]. During moxibustion the temperature at moxibustion site is variable within a range from 34°C to 57°C [16, 28–31]. However, is there an optimal temperature for moxibustion effect? In irritable bowel syndrome rat model [32], it demonstrated that 46°C, 50°C, and 54°C moxibustion at ST25 acupoint could obviously decrease the visceral hypersensitivity while 38°C and 42°C moxibustion groups did not show this effect, but there was no obvious difference among 46°C, 50°C, and 54°C. In acute hyperlipidemia, 46°C moxibustion on CV8 and ST36 acupoints could reduce the level of serum cholesterol, while 38°C moxibustion could not [33–36]. Another study indicated that 45°C moxibustion temperature could reduce serum IL-1*β* and TNF-*α* contents and raising IL-2 content, while 38°C moxibustion has not this effect [37]. The above-mentioned studies implied that the relationship between temperature and effect in moxibustion therapy should be a temperate-specific manner.

In this study, we found that moxibustion with 4 different temperature (37°C, 42°C, 47°C, and 52°C) obviously generated different analgesic effect in chronic inflammatory and neuropathic pain mice. It suggested that temperature would be really an important impact factor for moxibustion analgesia. We also found that the temperature-related moxibustion analgesic effect patterns were different on different pain model. In chronic inflammatory pain, the data presented that the higher the temperature of moxibustion, the better the analgesic effect. In neuropathic pain, the higher temperature (47°C or 52°C) of moxibustion produced stronger analgesic effect that lower temperature (37°C or 42°C), in which similar effect was displayed between 47°C and 52°C or 37°C and 42°C. Additionally, the peak time of analgesic effect was different between two pain models (inflammatory pain: 30 min versu neuropathic pain: 90 min).

Why the temperature-related moxibustion analgesic effect patterns were different on different pain model? The reason would be that CFA-induced chronic inflammatory pain model is sensitive to thermal stimulus [38, 39], while SNI-induced neuropathic pain model is much sensitive to mechanical stimulus [32, 40, 41]. That was why we employed TWL to access CFA-induced inflammatory pain model while we selected MWL to evaluate SNI-induced neuropathic pain model.

Current data inferred that in moxibustion therapy we should pay attention to not only the temperature but also different pattern of pain in pain management. In future study, clinical trial needs to be performed to confirm this finding from animal study.

## 5. Conclusion

In this study, it is suggested that the higher the temperature of moxibustion, the better the analgesic effect in chronic inflammatory mice; in neuropathic pain, the higher temperature (47°C or 52°C) of moxibustion produced stronger analgesic effect that lower temperature (37°C or 42°C), in which similar effect was displayed between 47°C and 52°C or 37°C and 42°C.

## Figures and Tables

**Table 1:** TWL of CFA mice (mean ± SE, s).

Group	*N*	Day 1 before CFA injection	Day 5 before moxibustion intervention	At 0 min after moxibustion	At 30 min after moxibustion	At 60 min after moxibustion	At 90 min after moxibustion	At 120 min after moxibustion
Control	18	15.26 ± 0.10	15.19 ± 0.15	15.22 ± 0.20	15.32 ± 0.09	15.36 ± 0.13	15.03 ± 0.13	15.38 ± 0.16
CFA	18	15.59 ± 0.09	3.74 ± 0.10^#^	3.87 ± 0.07	3.91 ± 0.05	4.09 ± 0.16	3.78 ± 0.09	4.17 ± 0.14
CFA-moxi-37°C	18	15.10 ± 0.2	4.17 ± 0.16^#^	5.63 ± 0.25^•^	6.52 ± 0.31^•^	6.18 ± 0.36^•^	5.32 ± 0.32^•^	4.67 ± 0.21
CFA-moxi-42°C	18	14.83 ± 0.25	3.84 ± 0.21^#^	6.67 ± 0.47^•^	8.05 ± 0.66^•^	7.53 ± 0.43^•^	6.54 ± 0.35^•^	5.17 ± 0.28
CFA-moxi-47°C	18	14.68 ± 0.17	3.68 ± 0.11^#^	8.74 ± 0.33^*∗*Δ•^	10.11 ± 0.42^*∗*Δ•^	9.22 ± 0.31^*∗*Δ•^	7.81 ± 0.51^*∗*•^	6.94 ± 0.30^*∗*Δ•^
CFA-moxi-52°C	18	14.78 ± 0.15	4.05 ± 0.11^#^	9.89 ± 0.31^*∗*Δ•^	14.37 ± 0.76^*∗*ΔΟ•^	12.26 ± 1.12^*∗*ΔΟ•^	13.57 ± 1.14^*∗*ΔΟ•^	13.05 ± 1.31^*∗*ΔΟ•^

^#^ *P* < 0.05 versus control group; ^•^*P* < 0.05 versus model group; ^*∗*^*P* < 0.05 versus CFA-moxi-37°C group; ^Δ^*P* < 0.05 versus CFA-moxi-42°C group; ^Ο^*P* < 0.05 versus CFA-moxi-47°C group.

**Table 2:** MWT of SNI mice (mean ± SE, g).

Group	*N*	Day 1 before SNI surgery	Day 5 before moxibustion intervention	At 0 min after moxibustion	At 30 min after moxibustion	At 60 min after moxibustion	At 90 min after moxibustion	At 120 min after moxibustion
Control	14	5.51 ± 0.04	5.29 ± 0.05	5.05 ± 0.12	5.11 ± 0.10	5.00 ± 0.09	5.20 ± 0.08	5.18 ± 0.08
SNI	14	5.50 ± 0.04	1.59 ± 0.03^#^	1.52 ± 0.04	1.60 ± 0.04	1.55 ± 0.04	1.60 ± 0.03	1.51 ± 0.02
SNI-moxi-37°C	14	5.58 ± 0.07	1.59 ± 0.07^#^	2.67 ± 0.30^•^	3.14 ± 0.13^•^	3.42 ± 0.14^•^	3.60 ± 0.26^•^	2.93 ± 0.25^•^
SNI-moxi-42°C	14	5.66 ± 0.09	1.43 ± 0.04^#^	2.38 ± 0.12^•^	2.98 ± 0.09^•^	3.21 ± 0.17^•^	3.56 ± 0.26^•^	2.80 ± 0.24^•^
SNI-moxi-47°C	14	5.41 ± 0.05	1.49 ± 0.02^#^	2.36 ± 0.06^•^	2.99 ± 0.08^•^	3.64 ± 0.08^•^	4.15 ± 0.15^•^	3.69 ± 0.60^*∗*Δ•^
SNI-moxi-52°C	14	5.56 ± 0.06	1.50 ± 0.02^#^	2.25 ± 0.04^•^	3.11 ± 0.07^•^	3.79 ± 0.05^•^	4.40 ± 0.04^*∗*Δ•^	3.66 ± 0.06^*∗*Δ•^

^#^ *P* < 0.05 versus control group; ^•^*P* < 0.05 versus SNI group; ^*∗*^*P* < 0.05 versus SNI-moxi-37°C group; ^Δ^*P* < 0.05 versus SNI-moxi-42°C group.
